# *In Vitro* Antiproliferative Effect of the Acetone Extract of *Rubus fairholmianus* Gard. Root on Human Colorectal Cancer Cells

**DOI:** 10.1155/2015/165037

**Published:** 2015-05-21

**Authors:** Blassan Plackal Adimuriyil George, Ivan Mfouo Tynga, Heidi Abrahamse

**Affiliations:** Laser Research Centre, Faculty of Health Sciences, University of Johannesburg, P.O. Box 17011, Doornfontein 2028, South Africa

## Abstract

Plants and plant derived products exert chemopreventive effects on various cancer cell lines by the induction of cell death mechanisms. The effects of root acetone extract of *Rubus fairholmianus* (RFRA) on the proliferation of human colorectal cancer (Caco-2) cells have been investigated in this study. The extract led to a dose dependent decrease in both viability and proliferation and increased cytotoxicity using trypan blue exclusion, adenosine 5′-triphosphate (ATP), and lactate dehydrogenase (LDH) assay. The morphological features of the treated cells were supportive for the antiproliferative activity. The Annexin V/propidium iodide staining indicated that *R. fairholmianus* induced toxic effects in Caco-2 cells and the percentages of the early and late apoptotic population significantly increased when compared with control cells. Also we studied the apoptosis inducing ability of the extract by analysing caspase 3/7 activity and the induction of cell death via the effector caspases was confirmed; the activity increased in treated cells compared with control. Thus the present findings highlight that the *R. fairholmianus* root acetone extract exhibits antiproliferative activity on Caco-2 cells by the induction of apoptosis via caspase dependent pathway.

## 1. Introduction

Cancer is a dreaded disease characterized by uncontrolled growth and spread of abnormal cells. The high mortality rate amongst cancer patients is an indication of limited efficiency of current therapies [1]. Identifying the mechanism of plant derived anticancer agents provides helpful information in cancer therapy. Natural products have been used for the treatment of various diseases for centuries. Evidences have shown that active principle compounds from plants may serve as potent chemotherapeutic agents with less toxicity to normal tissues and at low cost [2]. Plants have a long history of use in cancer therapy and it is significant that over 60% of currently used anticancer agents are from natural sources and around 80% of people in rural areas depend on plant products for their primary healthcare needs [3]. They are associated with induction of apoptosis, cell cycle arrest, inhibition of various signal transducers, and signaling pathways [4, 5]. Thus, it is important to screen the crude extract of plants or isolated compounds for their apoptotic potentials. The use of alternative medicine is increasing and many pharmaceutical industries are interested in developing plant derived medicinal compounds [6]. The increasing cost of conventional treatments and the lack of effective drugs encouraged people to depend more on folk medicine.

Berries such as* Rubus, Fragaria, Sorbus, Ribes,* and* Vaccinum* are common in Western diets. These soft fruits are rich in bioactive phytochemicals including several classes of phenolic compounds [7]. Berries from* Rubus* genus (cloudberry, raspberry, and blackberry) primarily contain ellagitannins and anthocyanins [8]. The evidence supports that anticancer properties of berries are due to the scavenging free radicals, induction of enzymes involved in xenobiotics metabolism, regulation of gene expression, alteration of cellular signalling, and induction of apoptosis [9, 10]. The search for better cytotoxic agents continues to be important in the discovery of modern anticancer drugs. Several bioactive compounds of plants act as lead compounds in drug discovery due to their structural diversity, bioactivities and the therapeutic potential of these compounds can be improved by molecular modifications.

Cancers that start in the cells lining inside of the colon and rectum are called colorectal cancers (CRC). CRC are the third most predominant cancers worldwide and are the fourth most common cause of cancer mortality with approximately 9.4% of global cancer cases irrespective of gender. The rate of incidence of CRC has been observed more in Australia/New Zealand, Europe, USA, and UK. African and Asian countries have a far lower rate of incidence [11]. According to epidemiological studies the major risk factors of CRC incidence include obesity, smoking, alcohol consumption, increasing age, family history, and dietary factors [12, 13]. Several flavonoid-rich foods could display relevant cancer-preventive effects. Thus, the isolation of potential chemopreventive agents from plants has contributed to breakthrough in anticancer studies [14]. Carcinoma colon (Caco-2) cell line originates from human colonic adenocarcinoma and shows similarities to small intestinal enterocytes [15]. These cells develop typical morphology of enterocytes with distinct apical and basolateral membrane domains, microvillus, and tight junctions [16]. The cells can be grown on uncoated polycarbonate filters to obtain well-differentiated monolayers [17].

The genus* Rubus *is very diverse, with over 750 species in 12 subgenera, and is found on all continents except Antarctica [18]. Due to useful ethnomedicinal and pharmacological properties,* Rubus* species have been used in folk medicine [19].* R. fairholmianus *(*R. moluccanus *L.) leaf extract has been administrated to reduce headache [20]; these leaves possess insecticidal properties; fruits are edible with stimulating effect [21]. On the basis of the previously reported, anti-inflammatory, wound healing [22], and* in silico* anticancer properties [23], we have selected this plant for this study. Even though this plant has immense ethnomedicinal value, a survey of literature revealed that the* in vitro *anticancer potential of this plant has not yet been evaluated. Keeping this in view, the aim of the present study was to investigate the* in vitro *anticancer potential and track the possible cell death mechanisms of* R. fairholmianus *root acetone extract, to put forward a scope to develop an effective drug.

## 2. Materials and Methods

### 2.1. Plant Material Collection, Identification, and Extraction

The fresh plant parts of* R. fairholmianus *were collected from Shola forest of Marayoor, Idukki District, Kerala, India, during September 2010. Plant parts were identified and authenticated (voucher specimen number BSI/SRC/5/23/2010-11/Tech.1657) by Botanical Survey of India, Southern Circle, Coimbatore, Tamil Nadu, India. The shade dried and powdered roots were extracted in Soxhlet apparatus using acetone. The extractability of phenolic compounds in the polar solvent like acetone was found to be high. Moreover, acetone extract has shown better pharmacological effects in many previous studies in* Rubus*. The extract was concentrated to dryness under reduced pressure in a rotary evaporator to yield dried root acetone extract. The dried extract then dissolved in 0.5% DMSO and was used for further analysis. The preliminary screening of leaves, stem, and root for total phenolics, tannin, flavonoids, and antioxidant properties showed that root had the maximum activity [24].

### 2.2. Cell Culture

The commercially obtained colorectal cancer cells (Caco-2 ATCC HTB-37) were used. Caco-2 cells were cultured in Dulbecco's modified Eagle's media (DMEM, Sigma-Aldrich, D 6429) with 1.2 g/L sodium carbonate, supplemented with 10% foetal bovine serum (FBS, Gibco, 306.00301), 10 mM nonessential amino acids (Gibco, 11140), 0.5 mM sodium pyruvate (Gibco, 11360), 2.5 mM L-glutamine (Gibco, 25030), 1% antibiotic (penicillin-streptomycin, Gibco, 15140), and 1% antifungal (amphotericin-B, Gibco, 104813) agents. Culture was maintained at 37°C with 5% CO_2_ and 85% humidity. Once cells reached 80–90% confluence, they were harvested and seeded at a concentration of 5 × 10^4^ in 3 mL culture media into sterile culture dishes, with a diameter of 3.4 cm. Cells were allowed to attach overnight.

### 2.3. Cellular Morphology-Inverted Microscopy

The effect of the plant extract on cell morphology was determined using an inverted light microscope (Wirsam, Olympus CKX41) after 24 h of incubation with different concentrations (5, 10, 20, and 40 *μ*g/mL) of RFRA and 0.5% DMSO. Once digital images were recorded, cells were trypsinized using 1 mL/25 cm^2^ of TrypLE Express (Invitrogen, 12605-028) and resuspended in Hank's Balanced Salt Solution (HBSS) to perform further assays.

### 2.4. Cell Viability-Trypan Blue Dye Exclusion Assay

The trypan blue dye exclusion assay (Sigma-Aldrich T8154) is a quantitative method to determine the percentage viability. Here, the viable cells with an intact cellular membrane do not take up the blue dye and maintain a clear appearance whereas the damaged nonviable cells take up dye and stained blue as their membrane damages.

The cell suspension was carefully mixed with 0.4% trypan blue reagent in 1 : 9 ratio and the mixture was transferred to a Neubauer hemocytometer to determine the number of viable and nonviable cells. Cells were counted by using automated cell counter (Countess Automated Cell Counter, Invitrogen).

### 2.5. Cellular Proliferation-Adenosine Triphosphate (ATP) Luminescent Assay

The CellTiter-Glo^1^ luminescent assay (Promega, G7571, Anatech Analytical Technology, Bellville, South Africa) is a homogeneous method for determination of cellular proliferation and quantification of ATP present in metabolically active cells.

Fifty microliters of reconstituted reagent was added to an equal volume of cell suspension and then mixed in a shaker for 2 minutes to induce cell lysis. This was then incubated at room temperature for 10 minutes in dark to stabilize the luminescent signal. The luminescent signal was read using the 1420 Multilabel Counter Victor3 (Perkin-Elmer, Separation Scientific).

### 2.6. Cytotoxicity-Lactate Dehydrogenase (LDH) Assay

The membrane integrity was assessed by estimating the amount of LDH present in the culture media. The cytosolic enzyme LDH will be released due to membrane damage. The CytoTox96 X assay (Anatech, Promega G 400) was used to measure the released LDH. Fifty microliters of reconstituted reagent was added to an equal volume of cell culture medium and incubated in the dark at room temperature for 30 min. The colorimetric compound was measured spectrophotometrically at 490 nm (Perkin-Elmer, VICTOR3).

### 2.7. Cell Death Analysis-Annexin V/PI Staining

The Annexin V fluorescein isothiocyanate (FITC) apoptosis detection kit (Becton Dickinson, 556570, Scientific Group, Randburg, South Africa) was used to detect apoptotic cells by fluorescence activated cell sorting (FACS). In apoptotic cells, the membrane phospholipid phosphatidyl serine, which is normally found in the internal portion of the cell membrane, becomes translocated to the outer leaflet of the plasma membrane, thereby exposing phosphatidyl serine to the external environment. Annexin V is a calcium dependent phospholipid binding protein that has an affinity for phosphatidylserine and is useful in identifying apoptotic cells.

Cells were resuspended in 1x binding buffer at a concentration of 1 × 10^6^/mL. One hundred microliters of the cell suspension was transferred to a 5 mL falcon tube and stained with 5 mL of FITC Annexin and 5 mL of propidium iodide (PI). The cells were gently vortexed and incubated for 15 min at room temperature and protected from light. After the addition of 400 mL of 1x binding buffer to each tube the samples were run in FACSAria flow cytometer (Becton Dickinson) by running 20000 events and analysed with CellQuest Software (BD Bioscience).

### 2.8. Quantification of Caspase 3/7 Activity

The effector caspases 3 and 7 play central role in apoptotic process. All steps were performed according to the manufacturer's procedure. Tumour necrotic factor-alpha (TNF-*α*) is able to initiate apoptosis by activating caspase 8, which in turn activates caspases 3 and 7. Activity of caspases 3/7 in apoptosis was determined with the Caspase-Glo 3/7 luminescent assay (Whitehead Scientific, Bracken fell, South Africa, Promega G8091).

Fifty microliters of treated cells was seeded in a 96-well luminescent plate (Scientific Group Adcock Ingram, Midrand, South Africa, BD354651). Fifty microliters of Caspase-Glo 3/7 reagent is added and the plate was then incubated at room temperature for 3 h. Luminescence was read using the Victor3 (Perkin-Elmer, Separation Scientific, Johannesburg, South Africa).

### 2.9. Statistical Analysis

A colorectal cancer cell line between 15 and 20 passages was used. Each set of experiments was repeated six times (*n* = 6) whereas each assay was performed in duplicates, with the results being averaged. Untreated cells were included throughout the course of study and all treated samples were compared to those cells by means of one-way ANOVA to determine the statistical difference. Statistical analysis was performed using SigmaPlot version 12.0 and the mean, standard deviation and standard error were obtained. Statistical significances between untreated control cells and treated cells are shown in the graphs as *P* < 0.05 (*∗*), *P* < 0.01 (*∗∗*), and *P* < 0.001 (*∗∗∗*). Significant differences were considered at the 95th percentile.

## 3. Results and Discussion

There are many reports available on the increased cancer cell death via induction of apoptosis or by many other means using different plant extracts or the natural compounds. Most of the chemopreventive agents used currently are nonspecific and kill both cancerous and normal cells causing various side effects. But the use of plant derived agents reduces this risk and it is more specific to the cancer cells. In this study the antiproliferative activity of* R. fairholmianus* root acetone extract on Caco-2 cells was examined.

**Figure 1:**
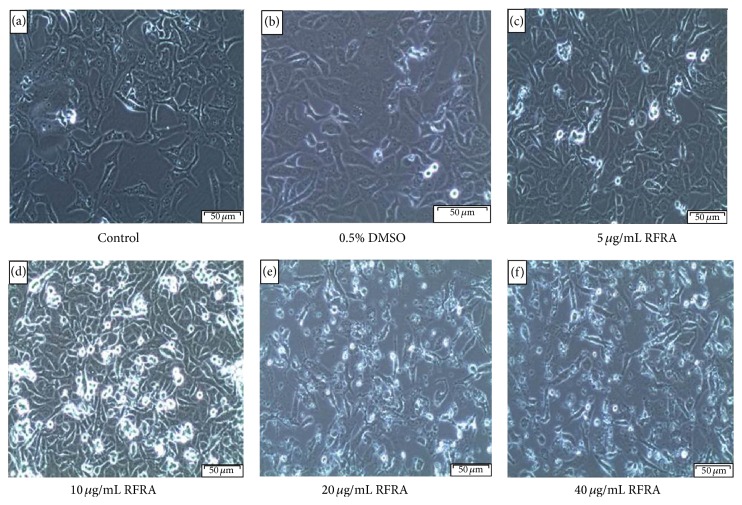
Morphological features of Caco-2 cells after 24 h treatment with RFRA (*R. fairholmianus* root acetone) extract. There were no significant visible differences in both control and 0.5% DMSO treated cells (a and b). The RFRA extract treated cells (c, d, e, and f) after 24 h showed loss of intact membrane, loss of contact with neighbouring cells, condensed and detached from the culture plate.

The morphological changes of Caco-2 treated cells with various concentrations of RFRA extracts were incubated for 24 h and compared with the untreated cells shown in Figure 1. Compared to control cells after the incubation period, morphology of RFRA treated Caco-2 cells significantly changed. The extract treated cells appeared less uniform with the loss of membrane integrity, although still intact at lower concentrations (5 and 10 *μ*g/mL). Whereas at 20 and 40 *μ*g/mL concentrations the RFRA treated cells showed remarkable difference with the control group. The significant changes such as loss of intact membrane, karyopyknosis, cell detachment from the plate, and change of morphological features were evident when compared to untreated cells. The most identifiable morphological features of apoptosis were observed by inverted light microscopy in the RFRA treated cells. The treated cells appeared like cells undergoing apoptosis with prominent features such as detaching from the culture plate, cytoplasmic condensation, cell shrinkage and condensation and aggregation of the nuclear chromatin, and loss of contact with neighbouring cells [25]. However the untreated cells appeared normal and were confluent.

The cell viability of the Caco-2 cells was assessed by trypan blue method. Trypan blue is an energy-dependent dye exclusion viability testing method; the dye is being excluded from live cells. As trypan blue is a weak acid, its affinity is increased for basic proteins; nuclei uptake is generally higher due to the presence of histones, yielding marked blue intensity, whereas the cytoplasm remains faintly stained [26]. This method helped to determine the percentage of viable and dead cells in the RFRA treated Caco-2 cells. The results are presented in Table 1. There was a dose dependent decrease in viability of the treated cells. The control cells showed 83.83% viability, whereas RFRA treated cells showed 71.33, 61.50, 52.50, and 41.00% for 5, 10, 20, and 40 *μ*g/mL, respectively. The decrease in percentage of viability becomes significant with all different concentrations of RFRA (*P* < 0.01 for 5 *μ*g/mL and *P* < 0.001 for 10, 20, and 40 *μ*g/mL, resp.). The net loss of viability at the higher concentrations in trypan blue assay indicated the cytotoxic effect of RFRA towards Caco-2 cells. No other studies have been published previously revealing the influence of RFRA on viability of Caco-2 cell lines. Similar results were also observed in* R. occidentalis* (black raspberry) extracts, they suppressed the proliferation of colon (HT-29), prostate (LNCaP), oral (KB, CAL-27), and breast (MCF-7) tumour cell lines [27, 28], and the cell viability decreased in a dose dependent manner. The extracts of some* Rubus* species such as* R. jamaicensis*,* R. rosifolius, R. racemosus*,* R. acuminatus*, and* R. idaeus *also exhibited great potential to inhibit cancer cell growth, inhibiting colon, breast, lung, and gastric human tumour cells [29].

The LDH leakage assay is a simple reliable and fast cytotoxicity assay based on the measurement of lactate dehydrogenase activity in the extracellular medium. LDH, a cytoplasmic enzyme released when the cell membrane damages, is assessed in cell culture supernatants [30]. The cell membrane damage of Caco-2 cells after the treatment with RFRA extract was measured by the release of LDH by following CytoTox96^1^ assay. The control cells and cells treated with DMSO showed lesser LDH release when compared to cells exposed to plant extracts. The RFRA treatments provoked a higher release of LDH activity than the control nontreated cells. There was a significant (*P* < 0.01 with 5 *μ*g/mL and *P* < 0.001 with 10, 20, and 40 *μ*g/mL of RFRA) dose dependent increase in the LDH release observed at increasing concentrations of RFRA (Table 1). The intracellular LDH release to the medium is a measure of irreversible cell death due to cell membrane damage, whereas Xia et al. [31] reported the direct involvement of LDH upregulation and subsequent induction of apoptosis.

**Figure 2:**
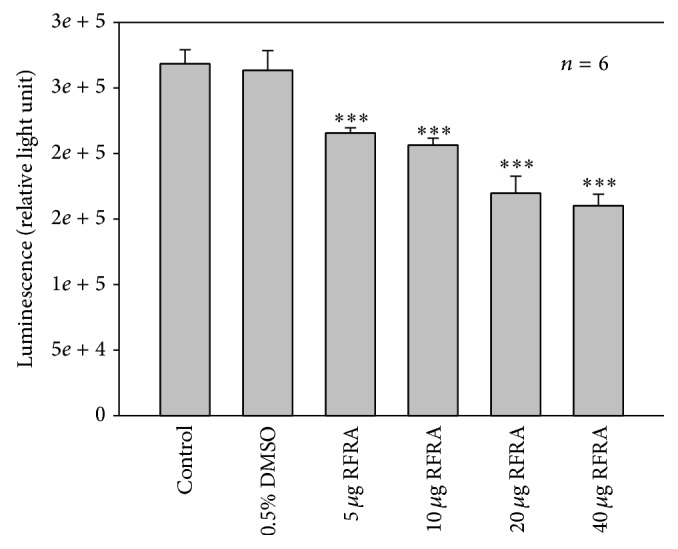
The ATP luminescent cell proliferation assay was used to determine Caco-2 cell proliferation. Control and 0.5% treated cells showed an increase in ATP level, whereas a dose dependent significant (*P* < 0.001) decrease in ATP level was observed in RFRA (*R. fairholmianus* root acetone) extract treated experimental groups (5, 10, 20, and 40 *μ*g/mL concentrations of RFRA).

Cellular ATP content was assessed to determine the level of metabolic activity of cells. The CellTiter-Glo^1^ luminescent assay was performed to evaluate the proliferating potential of cells. ATP is a marker for cell viability and proliferation and present in all metabolically active cells. Some reports suggest that ATP can induce cell proliferation, differentiation, and apoptosis, mediating different pathophysiological functions depending on the target cell [32]. The energetic level in Caco-2 cells remained higher, which was evident from the increased ATP level in control and DMSO treated cells. Exposure of Caco-2 cells to the RFRA caused a reduction in the intracellular ATP pool (Figure 2). The Caco-2 cells after RFRA treatments showed significant changes in cellular proliferation as compared to control cells. All the four tested concentrations of RFRA exhibited significant changes in cellular proliferation. The cells incubated with increasing concentrations (5, 10, 20, and 40 *μ*g/mL) of RFRA resulted in a dose dependent highly significant decrease (*P* < 0.001) in cellular proliferation compared with control Caco-2 cells. The actively dividing cells produce ATP, the main production occurs in mitochondria, and the depletion in ATP correlated with the decreased cell proliferation rate [33]. The ATP quantity drops soon after cell death and results in a loss of luminescence in assay [34]. RFRA extracts decreased the cell proliferation and was manifested from the decline of luminescence.

**Figure 3:**
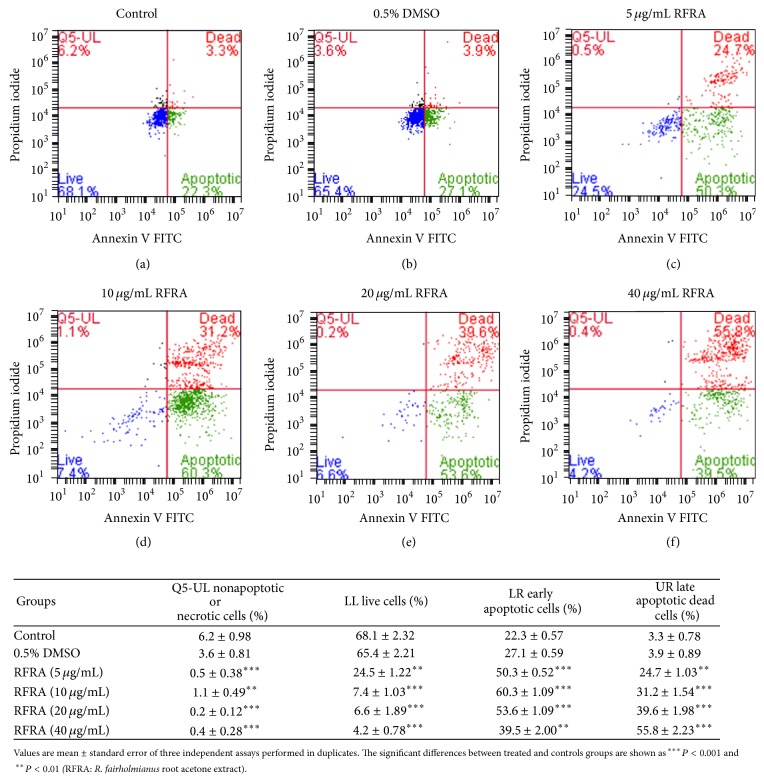
Annexin V FITC/PI staining used to assess the mode of cell death. RFRA (*R. fairholmianus* root acetone) treated Caco-2 cells showed an increased percentage of apoptotic population after 24 h incubation. The population of early and late apoptotic cells in control group found to be lower (22.3 and 3.3%) compared with experimental groups; the attached table shows the statistical data of flow cytometric analysis.

The Annexin V FITC apoptosis detection kit was used to distinguish the apoptotic and necrotic cells using flow cytometry. To further understand whether the decrease in cell viability observed was due to apoptosis, we examined the behaviour of cells after treatment using Annexin V/PI staining. The percentage of apoptotic cells increased with the increase in concentration of RFRA. The control and 0.5% DMSO treated cells showed no significant changes in percentage of cell population, most of the cells lined in the live cells range during flow cytometry analysis. The RFRA treated cells showed an increase in the percentages of early and late apoptotic cells. The nonapoptotic or necrotic cells concentration in the treated extracts was found to be very low compared with the control group.  Figure 3 shows the results of Annexin V-PI staining after the treatment with RFRA extract to Caco-2 cells. In the control groups, the Annexin V+/PI+ population was less (3.3%) and was compared with experimental groups. The RFRA treated groups such as 5, 10, 20, and 40 *μ*g/mL showed 24.7, 31.2, 39.6, and 55.8 percentages of late apoptotic cells population. When Caco-2 cells were treated with the RFRA, the early (Annexin V+/PI−) and late (Annexin V+/PI+) apoptotic population increased concurrently and induced a decrease in the viable cell (Annexin V−/PI−) and necrotic or dead cell (Annexin V−/P+) population. Although both late apoptotic and necrotic cells are Annexin V and PI positive, the presence of these cells with early apoptotic cells suggests that such dead cells resulted from the apoptosis rather than necrosis [35]. These findings confirmed that apoptosis may be the possible mechanism by which the RFRA triggers cell death in Caco-2 cells. The absence or significantly low percentage of cell population in quadrant (Annexin V−/PI+) of the RFRA treated Caco-2 cells ruled out necrosis [36].

**Figure 4:**
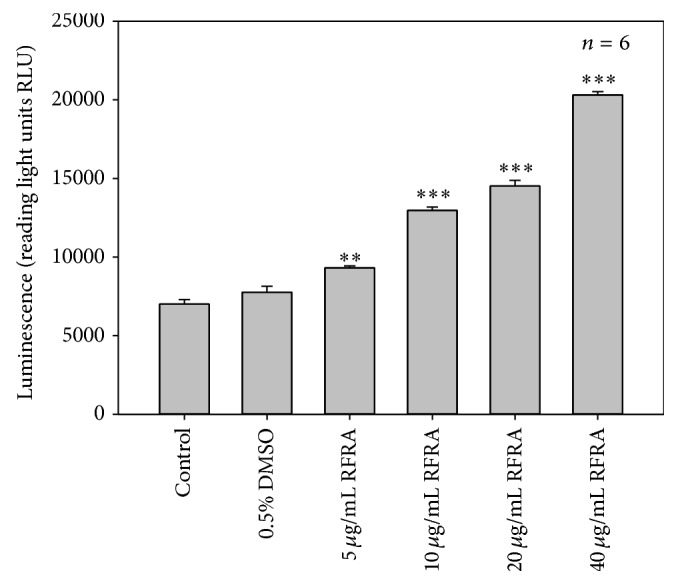
Caspase 3/7 activity determined as a function of caspase dependent apoptosis in Caco-2 cells after the treatment with RFRA (*R. fairholmianus* root acetone) extract. There was a significant dose dependent increase in caspase 3/7 activity after RFRA treatments (*P* < 0.01 and *P* < 0.001) compared to control and 0.5% DMSO treated cells.

To evaluate the inhibition of cell growth, release of LDH, the increase in early and late apoptotic cell population in response to RFRA treatments was due to caspase dependent pathway; the caspase 3/7 activity was measured. Caspases are a family of cysteine-aspartic proteases known as the vital mediators of apoptotic signalling [37]. Caspases 3 and 7 are well established as the major executioner (effector) caspases and their activation eventually leads to cell death [38]. The measure of caspase 3/7 activity in the cells is a direct mean for the determination of caspase dependent apoptosis. There was a significant dose dependent increase in the caspase 3/7 activities (*P* < 0.01 for 5 *μ*g/mL and *P* < 0.001 for 10, 20, and 40 *μ*g/mL) observed in treated cells with the increase in concentrations of RFRA (Figure 4). Low levels of caspase 3/7 activity were detected in the control and 0.5% DMSO treated cells, which was presumably due to the small amount of apoptotic cells present in the growing cell population. Among the effector caspases, caspases 3 and 7 play a vital role in the execution phase of intrinsic and extrinsic pathways of apoptosis by cleaving many crucial cellular proteins [39]. This cleavage facilitates disassembly of cell, which is evident from the morphological changes, such as cell shrinkage, chromatin condensation, membrane blebbing, and DNA fragmentation [40]. These morphological changes were also observed from the RFRA treated cells and strongly suggested the caspase mediated apoptotic cell death in Caco-2 cells. Kim and coworkers [41] reported the caspase 3/7 activity and apoptosis inducing ability of* R. coreanum*,a closely related species of* R. fairholmianus *on HT-29 human colon cancer cells.

Many plant extracts and isolated compounds have been reported for the antiproliferative activities and some of them showed their phototoxic effects on different cancer cell lines by inducing the cell death. Hypericin, a second generation photosensitizer isolated from* Hypericum perforatum*, has been shown to act as a potent light-activated photosensitizer in numerous* in vitro* and* in vivo* systems [42]. The further research is warranted to find out the photosensitising ability of* R. fairholmianus* since it has great antiproliferative activities. The irradiation of the extracts and isolated bioactive compounds may be helpful in killing cancer cells via generation of reactive oxygen species.

## 4. Conclusions

The present study demonstrated a dose dependent antitumor effect of* R. fairholmianus* root acetone extract on human colorectal cancer cell (Caco-2). A significant decrease in the cell viability, proliferation, and increase in cytotoxicity and caspase 3/7 activity were observed after the treatment with RFRA. The Annexin V/PI staining supports the apoptosis induced cell death. Moreover, the morphological alterations in the Caco-2 cells exposed to RFRA extract were suggestive for the apoptotic activities and this is described for the first time. The data demonstrate that this plant exhibits an inhibitory effect and seems to indicate the caspase 3/7 mediated apoptotic cell death triggered in Caco-2 cells. The outcome of this study recommends that the substantial antiproliferative activity of* R. fairholmianus *may be due to the caspase dependent apoptosis and the compounds of this extract may have promising use as a cancer chemotherapeutic agent.

## Figures and Tables

**Table 1:** Trypan blue viability and LDH cytotoxicity assays.

Groups	Viability percentage	LDH cytotoxicity
Control	83.83 ± 1.28	0.3490 ± 0.02
0.5% DMSO	82.83 ± 0.98	0.3677 ± 0.02
5 *μ*g/mL RFRA	71.33 ± 2.75^*∗∗*^	0.4580 ± 0.07^*∗∗*^
10 *μ*g/mL RFRA	61.50 ± 2.54^*∗∗∗*^	0.5328 ± 0.06^*∗∗∗*^
20 *μ*g/mL RFRA	52.50 ± 3.34^*∗∗∗*^	0.5943 ± 0.07^*∗∗∗*^
40 *μ*g/mL RFRA	41.00 ± 3.59^*∗∗∗*^	0.6508 ± 0.06^*∗∗∗*^

Values are mean ± standard error of six independent assays performed in duplicates. The significant differences between treated and controls groups are shown as ^*∗∗∗*^ *P* < 0.001 and ^*∗∗*^ *P* < 0.01. (RFRA: *R. fairholmianus* root acetone extract; LDH: lactate dehydrogenase; DMSO: dimethyl sulfoxide.)
